# From disease- to people-centred pandemic management: organized communities, community-oriented primary care and health information systems

**DOI:** 10.1186/s12939-023-02032-z

**Published:** 2023-10-23

**Authors:** Christine Leyns, Sara Willems, Richard A. Powell, Vivian Camacho, Ricardo Fabrega, Jan De Maeseneer, Salman Rawaf, Punam Mangtani, Austen El-Osta

**Affiliations:** 1 Research Institute of Social Sciences (INCISO), Faculty of Social Sciences, Universidad Mayor de San Simon, Cochabamba, Bolivia; 2 Department of Public Health and Primary Care, Faculty of Medicine and Health Sciences, Ghent University, Ghent, Belgium; 3 Department of Primary Care and Public Health, Faculty of Medicine, Imperial College London, London, UK; 4 High Level Commissioner at “Alma Ata 40 years High Level Commission for PAHO”, National Director inside Health Ministry in Bolivia, La Paz, Bolivia; 5 Dean, Faculty of Health Sciences, Universidad Santo Tomás de Chile, Santiago, Chile; 6 Department of Infectious Disease Epidemiology, Faculty of Epidemiology and Population Health, London School of Hygiene and Tropical Medicine, London, UK

**Keywords:** Community participation, Health information systems, Primary health care, Public health, COVID-19, Health equity

## Abstract

**Background:**

The COVID-19 pandemic exposed the health equity gap between and within countries. Western countries were the first to receive vaccines and mortality was higher among socially deprived, minority and indigenous populations. Surprisingly, many sub-Saharan countries reported low excess mortalities. These countries share experiences with community organization and participation in health. The aim of this article was to analyse if and how this central role of people can promote a successful pandemic response.

**Methods:**

This analysis was partly based on local and national experiences shared during an international and Latin American conference on person-and people-centred care in 2021. Additionally, excess mortality data and pandemic control-relevant data, as well as literature on the pandemic response of countries with an unexpected low excess mortality were consulted.

**Results:**

Togo, Mongolia, Thailand and Kenya had a seven times lower mean excess mortality for 2020 and 2021 than the United States of America. More successful pandemic responses were observed in settings with experience in managing epidemics like Ebola and HIV, well-established community networks, a national philosophy of mutual aid, financial government assistance, more human resources for primary care and paid community health workers.

**Discussion:**

Since trust in authorities and health needs vary greatly, local strategies are needed to complement national and international pandemic responses. Three key levers were identified to promote locally-tailored pandemic management: well-organized communities, community-oriented primary care, and health information systems. An organized community structure stems from a shared ethical understanding of humanity as being interconnected with each other and the environment. This structure facilitates mutual aid and participation in decision making. Community-oriented primary care includes attention for collective community health and ways to improve health from its roots. A health information system supports collective health and health equity analysis by presenting health needs stratified for social deprivation, ethnicity, and community circumstances.

**Conclusions:**

The difference in excess mortality between countries during the COVID-19 pandemic and various country experiences demonstrate the potential of the levers in promoting a more just and effective health emergency response. These same levers and strategies can promote more inclusive and socially just health systems.

## Resumen

**Antecedentes:**

La pandemia de COVID-19 expuso la brecha de equidad en salud dentro y entre países. Los países occidentales fueron los primeros en recibir vacunas y la mortalidad fue mayor entre las poblaciones indígenas, minoritarias y socialmente desfavorecidas dentro de los países. Sorprendentemente, muchos países subsaharianos reportaron un exceso de mortalidad bajo. Estos países comparten experiencias de organización y participación comunitaria en salud. El objetivo es analizar si y cómo este papel central de las personas puede promover una respuesta exitosa a la pandemia.

**Métodos:**

Este análisis se basa en parte en las experiencias locales y nacionales compartidas durante una conferencia internacional y latinoamericana sobre la atención centrada en las personas y comunidades en 2021. Además, se consultó los datos de exceso de mortalidad y los datos relevantes para el control de la pandemia, así como la literatura sobre la respuesta a la pandemia de países con un exceso de mortalidad inesperadamente bajo.

**Resultados:**

Togo, Mongolia, Tailandia y Kenia tuvieron un exceso de mortalidad promedio por 2020 y 2021 siete veces menor que los Estados Unidos de América. Se observaron respuestas pandémicas más exitosas en entornos con experiencia en el manejo de epidemias como el ébola y el VIH, redes comunitarias bien establecidas, una filosofía nacional de ayuda mutua, asistencia financiera del gobierno, más recursos humanos para atención primaria y trabajadores de salud comunitarios remunerados.

**Discusión:**

Dado que la confianza en autoridades y las necesidades en salud varían mucho, se necesitan estrategias locales para complementar las respuestas nacionales e internacionales a la pandemia. Se identificaron tres palancas clave para promover la gestión de pandemias adaptada localmente: comunidades bien organizadas, atención primaria orientada a la comunidad y sistemas de información de salud. Una estructura comunitaria organizada surge de una comprensión ética compartida que concibe a la humanidad interconectada entre sí y con el medio ambiente. Esta estructura facilita la ayuda mutua y la participación en la toma de decisiones. La atención primaria orientada a la comunidad incluye la atención a la salud comunitaria colectiva y las formas de mejorar la salud desde sus raíces. Un sistema de información de salud puede apoyar el análisis de la salud colectiva y la equidad en salud al presentar las necesidades de salud estratificadas por privación social, etnicidad y circunstancias de la comunidad.

**Conclusiones:**

La diferencia en el exceso de mortalidad entre países durante la pandemia de COVID-19 y las experiencias de varios países, demuestran el potencial de las palancas para promover una respuesta de emergencia sanitaria más justa y eficaz. Estas mismas palancas y estrategias pueden promover sistemas de salud más inclusivos y socialmente justos.

## Background

**Fig. 1:**
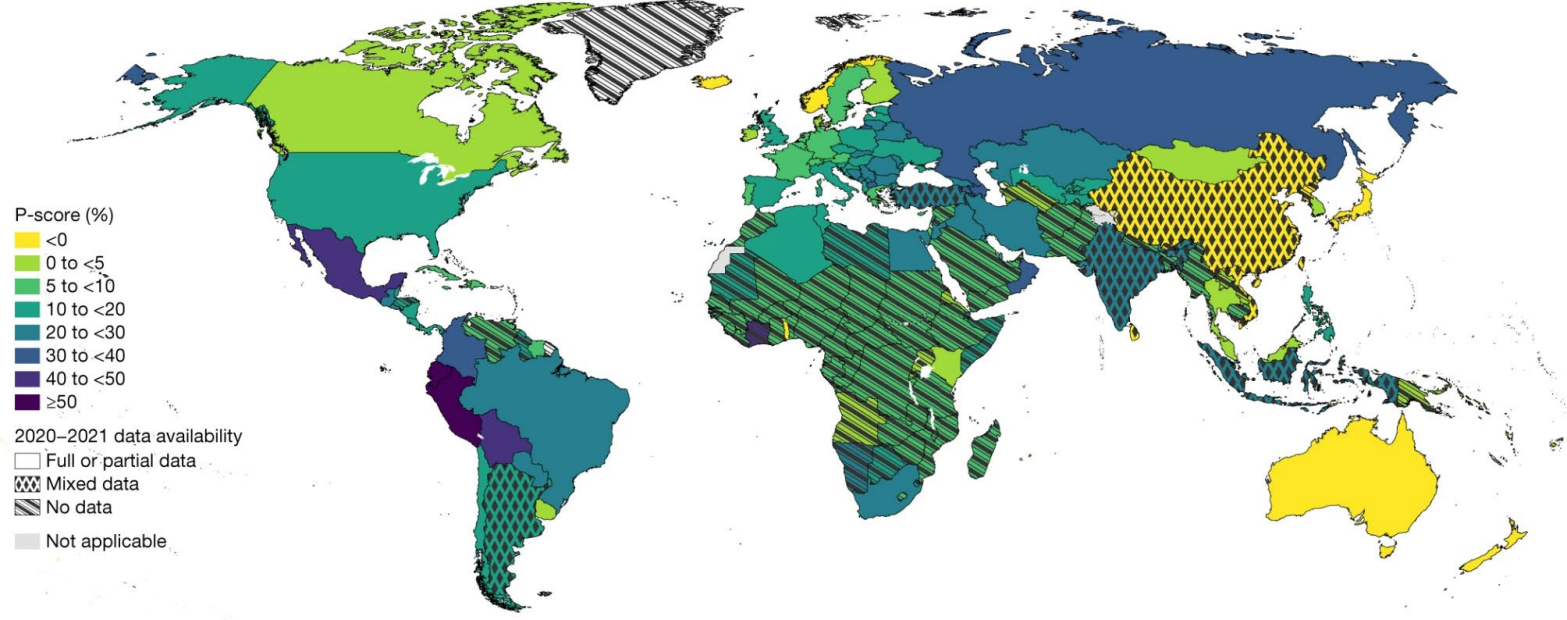
Mapping estimated P-scores (excess deaths relative to expected deaths) for years 2020 and 2021. The darker the colour the higher the estimated mean P-score. The patterns indicate the quality of the all-cause mortality data that were available for each respective country with the solid pattern showing full or partial data, dots for mixed data and diagonal lines for no data. (From: The WHO estimates of excess mortality associated with the COVID-19 pandemic) [4]

When COVID-19 reached people globally, a broad range of factors like age, chronic health conditions, living conditions as well as access to basic services, health care and information led to unequal levels of vulnerability. Indigenous populations, minority groups and people living in lower socio-economic strata presented higher rates of infection and mortality [1, 2]. Notwithstanding the universal widening of the pre-existing health equity gap [3], the difference in excess mortality between countries attributed to the pandemic in 2020 and 2021 is not, however, clearly associated with poverty as can be seen in Fig. 1.

Excess mortality measures both the direct and indirect impact of COVID-19 on vital statistics and is independent of the country’s test capacity. The P-score in Fig. 1 gives the ratio of the excess mortality to expected mortality for the years 2020 and 2021 expressed as a percentage. For Peru, the worst affected country, this P score is 97.1%, implying that nearly twice as many people as expected died during this period. By contrast, for Togo the P score was − 6.4%, which translates to 6.4% less people dying than expected [4]. The distribution of excess mortality on this map highlights factors other than wealth were related to a successful pandemic response. Effective mitigation was seen in countries that were not the richest, nor did they have better medical expertise.

In West African countries like Liberia, Sierra Leona, Nigeria and Togo, previous experience with managing infection outbreaks like Ebola may be related to their seemingly substantial lower excess mortality [5], although complete data are only available for Togo. Ebola control provided valuable lessons on the importance of trusted information sources to avoid fear and disbelief. Information passed on through community leaders, face-to-face communication, and through radios and trucks with speakers in the local language was perceived to be more credible than messages from national authorities. A strategy in the Ivory Coast applied a “monitoring committee” composed by selected community members including community health workers, religious leaders, traditional healers, and women and youth leaders, to implement “socially and culturally” appropriate Ebola virus disease prevention practices. Over 90% of participants reported compliance with most practices [6].

Both examples show how community-led disease prevention strategies backed up by appropriate consideration of socioeconomic and cultural factors stimulated community ownership and participation using people-centred health care, an approach that consciously adopts individuals’, carers’, families’, and communities’ perspectives as participants in, and beneficiaries of, trusted and resilient health systems that respond to their needs and preferences in humane and holistic ways [7]. Although putting people at the centre of their health is always necessary, since the health of a person depends more on life circumstances and life-style choices than on encounters with health care providers, this is even more pertinent in times of personal or collective healthcare crises [8].

A trusted healthcare practitioner can alleviate anxiety and stress by providing the best available medical evidence. This medical evidence, integrated with the contextual evidence provided by the people and policy evidence of public health, national and international authorities, can facilitate the co-creation of feasible health crisis mitigation strategies [9]. This article aims to analyse the impact of people-centred health strategies on the success of national pandemic responses and which levers may consolidate this potential.

## Methods

This analysis originated from the International 13th Geneva Conference on Person-Centred Medicine: Self-Care and Well-Being in the Times of COVID-19, 2021, and the Regional 7th Latin American Encounter on Person-Centred Medicine: Mutual and integral health care aimed at the wellbeing of all persons, conference on person- and people-centred care in times of pandemic. During these encounters, national and local experiences were shared that demonstrated the potential of person- and people-centred care, including mutual care. These cases were further explored in the literature and a table was created to highlight excess mortality during 2020 and 2021, as well as data that could have influenced this mortality in five different continents. Although quantitative data are included, this ecological exploratory study is mainly qualitative in nature, describing people-centred strategies applied in countries with a low registered pandemic excess mortality.

### Data

To be able to compare a variety of factors that can be related to excess mortality, 20 countries were purposively included. This inclusion was based on the level of excess mortality, the completeness of data stated by a recent WHO publication from January 2020 to December 2021 [4], the continent, gross domestic product (GDP) per capita and a minimum population size of 3 million inhabitants [10]. All cause monthly mortality data were available for only six out of the 47 countries of the Africa region (13%) with only three countries with data from January 2020 to December 2021: Togo, Kenya and South Africa. The only Southeast Asian country with complete monthly mortality data was Thailand. Three countries with data for less than the 24 months were included for reasons of comparison, being Canada, Algeria and Egypt [4]. No direct measure of civil society participation during the pandemic was found, making it necessary to search the literature for qualitative data for those countries included in the analysis. The closest indicator of social participation in policy found was “the deliberative component index”. This indicator [11] together with other indicators associated with pandemic control or excess mortality in the literature were included in Table 1.

- *Mean P-score* is the mean of the monthly ratio of excess deaths to expected deaths for 2020 and 2021, expressed as a percentage. For more details on how this ratio was calculated, see reference [4].
- *The population size for 2022 expressed in millions, and the GDP per capita for 2021 expressed in US$*, were collected from the world bank data [10].
- The *GINI index* or coefficient quantifies the degree of income or wealth distribution inequality on a scale from 0 to 1, with 0 representing perfect equality (where everyone has an equal share) and 1 representing maximum inequality (where one individual possesses all the income or wealth). In developing countries this metric overstates true income inequality since it dependent on reliable GDP and income data, ignoring informal economic activity [10, 12].
- *Cumulative vaccination uptake* as a percentage of the population by 1 January 2022 was obtained from Our World In Data [13]. This date was selected since by that point vaccines were available in all countries included.
- The *State Legitimacy Indicator* considers the representativeness and openness of government and its relationship with its citizens. The indicator looks at the population’s level of confidence in state institutions and processes, and assesses the effects where that confidence is absent, manifested through mass public demonstrations, sustained civil disobedience, or the rise of armed insurgencies. The value of the indicator varies from 0 to 10, with 0 a highly legitimate state and 10 means no state legitimacy [14, 15].
- *The Deliberative component index* is part of democratic government and associated with excess mortality [16]. It measures to what extent the deliberative principle of democracy is achieved, focusing on the process by which decisions are reached. A deliberative process is one in which public reasoning focused on the common good motivates political decisions. The index is formed by point estimates drawn from a Bayesian factor analysis model that includes the following indicators: reasoned justification, common good justification, respect for counterarguments, range of consultation, and engaged society [11].

## Results

Countries in this analysis include western countries from Europe and North America, as well as countries in Asia, Africa and South America. Table 1 presents the countries ordered from lowest to highest excess mortality. It shows how countries like Togo, Mongolia, Thailand, and Kenya, with a GDP more than ten times lower than the United States of America (USA), reported a mean P score for 2020 and 2021 that was seven times lower. The Western countries with the highest mean excess mortality were the United Kingdom (UK) and the USA. Vaccination uptake by the end of 2021 was lowest in the African continent, the percentage of the population that received at least one vaccine varied between 10.98% in Kenya and 31.63% in South Africa. In Uruguay and Norway trust in the government was high, expressed by a state legitimacy indicator below 1. For Bolivia, Peru and Chile, as well as for the UK and the USA, this trust was much lower.

**Togo** had no apparent excess mortality while it had the lowest GDP per capita, the highest GINI index, a low vaccination uptake and low state legitimacy. The country adapted an early digital public assistance relief which increased households’ trust in the government’s crisis management approach [17]. Other factors that may have influenced the pandemic response were experience with Ebola outbreak preparedness like in other West African countries [5], and the African philosophy of mutual aid, described for **Kenya**. Although, two months into the outbreak, the Kenyan government had not yet met its promise to provide a weekly stipend to vulnerable households across the country, social networks proved an important source of social protection. The pandemic motivated many Kenyan families to come together, share food and other resources, convey health information, care for children and the sick or elderly and provide mental and emotional support. This collaboration is emblematic of Kenya’s harambee spirit, a culture of people uniting in times of need that goes back to the country’s post-independence nation-building efforts. Community networks have been key in identifying vulnerable households and ensuring that those who need help access it [18]. Kenya has a strong network of mentors and peer educators for HIV prevention, comprised of trusted individuals within vulnerable communities. In the COVID-19 crisis, these networks have shifted gears to battle coronavirus misinformation through door-to-door advocacy and, in a nod to social distancing, small peer-to-peer groups [19].

**Thailand** adopted a whole-of-society approach whereby citizens, the private sector and civil society worked together to mitigate the impact on vulnerable populations. Strong social capital was demonstrated by a voluntary “food pantry” initiative, through which individuals, communities, temples, and mosques filled and refilled food and essential items into community-based “pantries” for those in need. This societal fabric and the spirit of helping others reflected the generosity and hospitality seen among Thais. Additionally, Surveillance and Rapid Response Teams working at the local level were complemented and supported by 1.04 million village health volunteers in communities. Since 2009, each volunteer has received a monthly honorarium for their work, adjusted in 2019 and increased with 50% during the COVID pandemic [Tangcharoensathien V, Vandelaer J, Brown R, Suphanchaimat R, Boonsuk P, Patcharanarumol W. Learning from pandemic responses: Informing a resilient and equitable health system recovery in Thailand. Front Public Health. 2023 Jan 25;11:1065883. doi: 10.3389/fpubh.2023.1065883. PMID: 36761120; PMCID: PMC9906810].

**Mongolia** has a female literacy rate of 96.4% and 2.5 million people use mobile telephones, of which over 70% are smartphones, facilitating public education and the dissemination of public messages. There are approximately 12,000 doctors and more than 20,000 mid-level health workers, of which more than 12,000 are nurses, resulting in one physician and one nurse for 283 citizens. The State Emergency Committee initiated a one-window policy to provide accessible and reliable information daily at a set time through all communication channels and media. The Ministry of Health has sent frequent text message alerts nationwide with recommendations on avoiding unnecessary domestic and international travel, self-isolation for incoming travellers, nutritional advice, and personal hygiene and protective measures [20].

**Uruguay** was the most successful country in the South American region in controlling viral spread through high levels of community involvement combined with at-scale coronavirus testing. Its government invested in public information campaigns and primary health care [21].

**Table 1:** The mean ratio of the excess mortality to expected mortality for the years 2020 and 2021 with factors that can have influenced mortality

Country	Mean p score (%)^1^	Population 2022 (MM)^2^	GDP per capita 2021 (current US$)^2^	GINI^2^	Vaccination uptake by December 2021^3^	State legitimacy 2020^4^	Deliberative component index 2020^5^
Togo	**-6.4**	8.8487	973.21	42.4	15.73	8.5	0.86
Japan	**-0.7**	125.12499	39,312.66	32.9	81.66	0.6	0.91
Norway	**-0.1**	5.45713	89,154.28	27.7	78.99	0.5	0.99
Mongolia	**0**	3.39837	4,566.14	32.7	66.68	3.8	0.80
Thailand	**1.5**	71.69703	7,066.19	35.1	71.71	7.6	0.13
Kenya	**2.1**	54.02749	2,081.80	40.8	10.98	7.9	0.87
Finland	**2.5**	5.55688	53,654.75	27.1	77.16	0.6	0.93
Canada	**3.8**	38.9299	51,987.94	32.5	82.58	0.5	0.89
Uruguay	**4.4**	3.42279	17,313.19	40.8	80.64	0.5	0.86
Belgium	**7.7**	11.66945	51,247.01	26.0	76.72	1.1	0.93
United Kingdom	**12.0**	66.97141	46,510.28	32.6	76.71	2.9	0.78
United States of America	**15.1**	333.28756	70,248.63	39.7	73.53	2.9	0.69
Algeria	**16.5**	44.90322	3,690.63	27.6	15.72	8.3	0.49
Chile	**17.2**	19.60373	16,265.10	44.9	88.29	5.7	0.90
Egypt	**21.5**	110.9901	3,698.83	31.9	29.82	8.6	0.33
South Africa	**22.7**	59.89389	7,055.04	63.0	31.63	6.2	0.90
Kazakhstan	**28.8**	19.62197	10,373.79	27.8	46.35	8.5	0.34
Plurinational state of Bolivia	**48.8**	12.22411	3,345.20	40.9	46.28	7.5	0.49
Ecuador	**50.6**	18.001	5,965.13	45.8	79.02	6.0	0.67
Peru	**97.1**	34.04959	6,621.57	40.2	72.98	7.1	0.72

^*1*^*From: The WHO estimates of excess mortality associated with the COVID-19 pandemic* [4]; ^*2*^*Data World bank* [10]; ^*3*^*Our World in data* [13]; ^*4*^*Fragile states index* [15]; ^*5*^*Varieties of Democracy* [11]

## Discussion

Strong community networks experienced in health education, a local philosophy of mutual aid, communication between health authorities and the population, sufficient human resources at the primary health care level, trust in the government and financial support seem to be factors present in countries that reported low mean excess mortality rates for 2020 and 2021.

In the literature, an increase in democratic governance [16] and in community involvement in decision making [13, 21, 22] were associated with a decrease in excess mortality, whereas higher levels of trust in government were associated with a higher compliance with proposed COVID-19 preventive measures [22, 23].

A strength of this study is the ability to use all-cause deaths compared to previous years to assess the impact of COVID at the ecological level, albeit this was based on the assumption that registration of deaths remained the same notwithstanding the social disruption seen in countries with the highest excess mortality. Limitations of this ecological study are the incompleteness of the countries that could be included and the lack of an in-depth description of the pandemic response per country, making it necessary to interpret the findings with caution. The inclusion of all possible confounding factors falls outside the scope of this study, which aimed to describe the elements related to people-centred care. For some countries like Togo, even related to this strategy, very little has been published and an in-depth analysis would be an important contribution to existing knowledge.

### Tailored local people-centred strategies

The experience in Togo, Kenya, Mongolia and Thailand suggests that in country settings or populations where trust in the government is low, local strategies, involving local leaders and understanding local beliefs, can help address rumours and misinformation [24, 25]. The impact of the absence of such strategies can be seen for Peru, Ecuador and Bolivia, leading to high levels of excess mortality amidst a socio-political crisis and initial neglect for the role of primary health care [26, 27]. Local strategies are especially relevant for socio-culturally diverse populations, or in times of internal conflict or war as well as for specific vulnerable populations within countries.

A similar strategy was used in Ireland with Irish Roma, a population distrustful to the rest of society, and traveller groups’, people without a fixed abode, organizations. The goal was to minimise the widening of existing health inequity through a community-health partnership with primary healthcare professionals trusted by these populations. Beside communication tailored to the culture (norms, beliefs, and values) and literacy needs, they successfully advocated for public health measures like access to water and sanitation, financial and logistic support and prioritized access to COVID-19 testing. This project levelled access to health care and reduced COVID-19 exposure in this population [25].

The potential of involving people in reducing the impact of a health crisis calls for a debate and a scientific assessment on the need to integrate contextual knowledge or the people´s knowledge systems in health service and health system management [9]. If we look at a health problem like COVID-19 with a narrow medical lens, the health sector creates an expensive best-care practice for individuals, driven by pharmaceutical actors that see the pandemic as an opportunity for short-term profits [28]. This is neither affordable nor acceptable for a large share of the world’s population. A start is being made for inclusive initiatives within the Coalition for Epidemic Preparedness Innovations (CEPI), a global partnership between public, private, philanthropic, and civil society organisations launched in 2017, to develop universally accessible vaccines and other biologic countermeasures against epidemic and pandemic threats [29].

### Interconnectedness versus individualism

The strict respect for individual freedoms and rejection of measures focusing on the common good in western countries is particularly ill-suited to the management of global threats. Individualism leads to divergent views, including the disruption of social norms in countries like the UK, Canada, Australia, and the USA. The absence of a shared social ethic weakens communal bonds and impacts individual stress, frustration, anxiety, confusion, and powerlessness [30]. Insight into the unsustainability of the prevailing individualism of Euro-American countries, where the individual improves his own life with little regard for the future, is growing. The more individualistic people were, measured by the weight they give to their personal interests rather than their in-group’s interest, the higher the chances they would not adhere to epidemic prevention measures [31].

The need to put interconnectedness central in health was identified during the High-Level Commission, ‘40 years of Alma Ata’, with the term “mutual care”. This concept includes, beside people as individuals, families and communities, the health and social care workforce, and the environment at large [32]. This wisdom on the balance of a person with others and their environment originates from ancient cultures across the globe. The African concept Ubuntu, the essence of being human, translated incompletely as “in existing with and through others”, reflects interconnectedness in the present, the past and the future; my life affects not only now but also in the future the lives of others [33]. In Bolivia, this is described through the words “to live good” *(“buen vivir*”), a translation from the Aymara expression *“Suma Qamaña”* or *“Sumaq Kawsay”* in Quechua *or “Ñande Reko”* in Guarani, a literal translation of “being whole”, to live with beauty and harmony. The well-being comes from the understanding that our existence is related to the world; we do not only depend on Mother Earth or Pachamama but are also part of it. For the *Sumaq kawsay* the relationship and balance an individual has with the community and the natural environment is fundamental. They therefore urge communities to organize themselves and function in a way that the whole community benefits, to achieve a satisfactory life, to promote community life, sharing and caring for each other as part of life systems that promote reciprocal care. The Pachamama (mother earth) is not seen as a resource to use without limits but as a living creature with whom we must learn to live in harmony; if we destroy our environment, we destroy ourselves. Healthy food, healthy water, and healthy air are the main natural remedies that preserve human health; if Mother Earth is healthy, we are healthy. “To live good” means being complementary and living together in solidarity without excessively competing with one another and the natural environment [34].

Interconnectedness has been the basis of many pandemic experiences in Latin America. In Chile, the *Journal of the Association of Family Physicians (Revista Chilena de Medicina Familiar)* published a special issue on person- and community-centred experiences [35]. In Mexico, this experience is reflected in “self-help groups”, where people share their strategies and knowledge in the management of their diseases, with a proven benefit to treatment adherence and user satisfaction [36]. In Peru, the oldest and best-documented experience is the program “the life reform” and the health circles from the largest social health security system (EsSalud), where some workers in median and big companies were formed as health promotors to supervise and promote a healthy life for their colleagues [37].

### Proportionate Universalism through community oriented primary care

People have a central role in disease prevention and health promotion through self-care backed up by adequate health literacy and a supportive environment [38]. As such, the initial solution proposed in many countries, predominantly virus- and biomedically-focused, like new pharmaceutical products, vaccines, and national Test-and-Trace programmes, was incomplete. It de-emphasised the diversity between people, their organization and their potential in helping tackle the pandemic. Building structures that promote continuous community engagement to mitigate current and future healthcare crises, including lifestyle diseases, requires a health system based on primary healthcare. A primary healthcare organized in a way that facilitates a structural collaboration between the population and the healthcare team [39]. This was proposed in Alma Ata [40] and piloted in the framework of Community Oriented Primary Care (COPC) since the 1940s in South Africa, where its development was hindered during the height of apartheid [41]. The initial global pandemic directions underestimated the role of primary care as a trusted information source and as an expert in local social and health needs [42].

For COPC to be effective there are preconditions related to the communities as well as to the health care organization. To work with and engage communities, legitimate representatives and a clearly defined goal facilitate engagement. These representatives can form a **health committee** at the level of the primary care service to analyse prominent health problems and identify how to address their underlying causes [43]. The primary healthcare and community-driven health pathways need collaboration and coordination within the health sector, and with other sectors like social services, employment, education, and basic services like water and sanitation. At the primary care level, a clearly defined practice population and incentives for community engagement, health promotion and disease prevention are necessary. Putting primary health care and communities central to future health care crises can facilitate a locally tailored response that takes the level of need or disadvantage in a population into account [44].

Public health and primary care play a central role in tailoring the pandemic response to people´s needs [30]. Understanding health related to social, cultural, economic and environmental factors is crucial. These factors are known as the social determinants of health, and they provoke the unfair and avoidable differences in health status seen within and between countries [44]. Ignoring these factors through a one-size-fits-all isolated health system and disease-focused response is unlikely to be effective nor inclusive when attempting to cope with a public health emergency. Social determinants of health tended to deteriorate during the pandemic, including early childhood development, education, food security, social inclusion and openness to diversity [45]. A refocus on community-oriented primary care is crucial as it takes these factors into account. Involving well-informed communities and their social determinants in pandemic preparedness and rapid response strategies is essential to reduce, or at least not exacerbate, existing health inequities [46].

### The role of technology to support public health, people, and health services

Technology can facilitate the identification of vulnerable populations, social determinants of health and health problems through data measurement and sharing. If equity is not explicitly addressed, healthcare responses can unwillingly exacerbate health inequalities.

In 2008, health care improvement was described by Berwick and colleagues as the triple aim: improving population health, enhancing the care experience, and reducing costs, where the aims reinforce one another [47]. In 2014 a fourth aim, avoiding burnout of health care providers, was added as an essential component, and in 2022 the fifth aim, advancing social justice and inclusion. If this last aim is not considered, the risk of standard improvements like an uptake of preventive care through alerts in the electronic health record, can increase disparities for people who do not access care. Actions around the fifth aim could be partnering with community-based organizations to organize screening and vaccination for non-responders of national call and recall systems, working with community health workers to make health education more relevant and acceptable, and communicating strategies to address specific concerns and transportation provision to facilitate access [48].

To tackle health inequity it is essential to invest in its measurement as well as in data sharing between health care and community-based organizations. Data stratified by relevant social categories, including self-reported race, ethnicity and gender identity and data on social needs, and barriers to care, such as transportation, food insecurity and housing [48], assisted in pandemic management. In the UK these data showed how pre-existing racial and socioeconomic inequalities exacerbated COVID-19 health outcomes for ethnic minority populations [1], whereas in Belgium these data enhanced equity by facilitating the organization of a priority-vaccination for those most at risk [49].

Inevitably in many countries, technology, vaccine development and treatment trials received the lion´s share of economic and human resources, at the cost of people-centred strategies and health equity [7]. The singular focus on curative, disease-centred and health service-focused approaches and the under-use of community-centred approaches meant that health systems in many countries remained under insurmountable pressures and some, like Brazil, Peru and Bolivia, eventually collapsed [50]. Conversely, other countries such as Uruguay, invested successfully in less costly public information campaigns and primary health care [21].

A comparison of how primary health care is organized in different countries, including how it adapts to local needs and interacts with the community, through health committees, community health workers or with individuals, needs to be further explored. Elements related to the impact of community-centred approaches during a healthcare crisis need to be studied at the local level. In a global world, individualistic and interconnected population groups are present in most countries in the north and the south, making it difficult to interpret the effect of social cohesion and strategies aggregated to a national or even regional level.

## Conclusions

A reflection on the traditional wisdom present in African, Asian and Latin American heritages can bring the complexity of life and health back to its essence: our interconnectedness. If humanity is rebuilt around the principle that living well means the wellbeing of all people and the environment in the present and in the future, power relations will be more just, trust will grow, and fear will diminish. The empowerment of communities can promote resourcefulness and agency and eventually strengthen self-reliance capabilities in the most resource-poor or unhumanitarian settings, as well as in western settings where loneliness, social isolation and poor mental health are highly prevalent.

The COVID-19 pandemic challenged health systems worldwide causing a humanitarian and societal crisis, but also presented an opportunity to reflect on how to build back better. A top-down, global policy-driven, disease-focused response, including over-reliance on national lockdowns has largely undermined a comprehensive approach that takes the different vulnerabilities and capabilities of people into account. The interconnectedness of people, primary health care and the documentation and analysis of social factors are key levers to tackle future pandemics. For a global problem, resources must be aligned for a global goal: better health care for all. If health equity is not measured and addressed as a goal on its own, health system responses and advances tend to widen the health equity gap. Primary health care oriented towards both individuals and communities, supported by adequate data registration and sharing can together with the community monitor health equity, co-design strategies to address inequity and support its implementation. A country which fully encourages primary healthcare, equity measurement and community organization facilitates the integration of bottom-up resources with top-down measure, and in so doing, it complements comprehensive person-centred approaches and mass campaigns with targeted strategies to lever pandemic preparedness as well as health equity.

## Data Availability

Figure 1 is part of a WHO article licensed under a Creative Commons Attribution 4.0 International License. The complete article can be consulted via this link: The WHO estimates of excess mortality associated with the COVID-19 pandemic | Nature. The datasets used to generate Table 1 are from The WHO estimates of excess mortality associated with the COVID-19 pandemic | Nature., indicators of the world bank, https://data.worldbank.org/indicator, Our World in Data, https://ourworldindata.org/coronavirus, open access under the Creative Commons BY license, The Fund for Peace. Fragile States Index available from: https://fragilestatesindex.org/about/ and the Varieties of Democracy (V-Dem) project available from: https://v-dem.net/about/v-dem-project/.

## Abbreviations

COPC: Community Oriented Primary Care
UK: United Kingdom
USA: United States of America
